# Chicago Medical Society, July 6, 1874

**Published:** 1874-07-15

**Authors:** Will. T. Montgomery


### Society Reports.

### CHICAGO MEDICAL SOCIETY.

### REGULAR SEMI-MONTHLY MEETING JULY 6, 1874.

*Reported by Will. T. Montgomery.*

THE Chicago Medical Society met, as usual, in the parlor of the Gault House. The President, Dr. Quine, in the chair. The Secretary being absent, Dr. Graham was elected Secretary *pro tem.* Dr. Strong related the following case: A young lady, nineteen years old, had been well until about one year ago, when she began to feel a stiffness in the calves of her legs. At that time she had to walk a considerable distance in order to reach her school, and thought at first that the trouble arose from this circumstance, but soon inflexibility and contraction of the joints supervened, and locomotion became impossible. Dr. Strong first saw her about four months ago. He found a large, flabby, and fleshy patient, with legs firmly flexed upon the thighs, but with no bony anchylosis of the knee joints. By persistent effort he was able to extend the legs to an angle of about forty-five degrees. He was not able to find the tendons of the hamstring muscles, but found a firm cord in the centre of the popliteal space, which appeared to be composed of the united tendons. There was considerable œdema of the feet and legs, which seemed due to the dependent position. Sensibility was perfect. The patient had not had any pain, and no tenderness existed along the spine. Appetite and sleep were normal, and she stated that she felt otherwise well. Menstruation had begun at sixteen years of age, and had regularly continued up to two months before the date of examination. Tonics, stimulating liniments, and passive motion were ordered, but with no improvement of symptoms. Dr. Mary Thompson suggested pelvic cellulitis as the probable cause of the trouble. The President thought it probably depended upon some lesion of the lower portion of the spinal cord. Dr. Strong thought, if the trouble had come from cellulitis, the patient would have experienced pain. The President referred to a case of cellulitis in which there had been very little pain. Dr. Stillians reported the case of a young lady twenty-one years old, who had been sick since she first began to menstruate, seven years ago. She had been under treatment most of this time, but he did not see her until within the last ten weeks. He found her apparently well nourished, but complaining of pain and stiffness in one knee, and general hyperæsthesia of the integumentary surface. She was vomiting, and able to retain certain kinds of food only. He prescribed anti-emetics and extended the limb, but finding this treatment inefficacious, he began, three weeks ago, the use of tonics and electricity. The vomiting had ceased and the patient had been able to walk, but had had a hacking cough with anorexia for the last four days. The speaker was disposed to consider the ailment hysterical. Dr. Paoli agreed with the latter opinion, and recommended the use of assafœtida and valerian.

Dr. E. F. Ingals reported a case of epileptiform neuralgia with tonic muscular spasms. He had been called about midnight, June 24, to see the patient, who was about twenty-one years of age. She had been suffering from severe cramps of the arms, legs, and stomach for three or four hours. He found the patient in bed, apparently suffering but little, and was told she had improved much within an hour. Upon inquiry, he learned that she had been suffering from occipital neuralgia of a not very severe type for several days, the pain commencing late in the afternoon and continuing three or four hours. The patient stated that during the previous afternoon, the pain had been more severe than usual, and had been succeeded by chilly sensations of the feet and legs, followed by cramps, affecting also the hands and, finally, the stomach. Upon examination he found the skin cool and moist, the pulse slightly accelerated, the pupils normal, and the tongue slightly coated. No vomiting nor purging had occurred, though the bowels had been evacuated with paroxysmal abdominal pain. He prescribed one-quarter of a grain of the sulphate of morphia and ten grains of hydrate of chloral, to be repeated once in three hours if the convulsions returned. The next day moderate cephalalgia and muscular soreness remained. The spasms did not return during the night, but the patient slept little. About a year ago the patient had occupied a damp basement with poor sewerage, from which she had been finally induced to remove, though not until an obstinate intermittent neuralgia, for which she had been treated, had yielded. At that time quinine and iron utterly failed to give relief, which was afforded by the use of granules of one-sixteenth of a grain of sulphate of strychnia, given three or four times daily. Subsequently she had enjoyed good health until her present illness. Careful inquiry revealed the fact that the old attack had been preceded by tonic convulsions similar to those of the present illness. Remembering the former treatment, he at this second visit, notwithstanding the convulsions, prescribed the strychnia in granules of one-sixteenth of a grain each, four times daily. That evening, about 5 o'clock, convulsions of the extremities and maxillary muscles again occurred, and lasted about three hours. He called at 5 P. M. the following day, and found the patient feeling very well, without headache and pain, and with some appetite. He congratulated himself that convalescence had been established, but in about half an hour after his visit the convulsions returned in a severe form. When examined at 9 P. M. the hands alone were affected, the fingers being firmly flexed by tonic muscular contraction. Suspecting that the medicine had occasioned the relapse, he instituted inquiry and found the druggist had made pills instead of furnishing the granules ordered. Morphine and chloral, as before, were substituted for the styrchnia, and on the 27th inst., he ordered half a grain of the valerianate of zinc at a dose, made into a pill with confection of roses to be given three times daily. Convulsions milder than before, occurred that evening. Two days later he found the patient comfortable. She had escaped pains and cramps the previous evening. What was the cause of this sudden cessation of neuralgic pains and the accompanying convulsions? "The valerianate of zinc" would be the answer, he thought, of nine out of ten physicians; but he found that the patient had not taken this medicine, owing to a misunderstanding on her part. A few doses, however, had been taken, but not sufficient to account for the relief. Two days later he found her still improving, and not suffering from convulsions. He ordered the medicine continued for a day or two in doses of one grain, to be followed by a ferruginous tonic. The doctor remarked that the case seemed interesting, in the first place on account of the convulsions attending an otherwise simple case of intermittent neuralgia. The first question occurring to us is: what caused the convulsions? Doubtless the same cause operating upon the cerebro-spinal axis produced convulsions which had formerly caused occipital and abdominal neuralgia, but what the exact nature of this cause was, he was unable to say. The patient was neither of a rheumatic nor gouty diathesis. She had not been exposed to lead poisoning, and could not be properly called anæmic. The pains were distinctly intermittent, and so one might expect malaria. But when we remember that many nervous affections, not dependent upon malaria, exhibit an intermittent character, we are still left in doubt. He said it seemed to him as unphilosophical to call every intermittent affection malarial, as to commit the common blunder of calling every disease rheumatic or syphilitic from which the patient recovers while taking iodide of potassium.

In the next place he wished to call attention to the use of sulphate of strychnia in doses of one-sixteenth of a grain. Whether in this instance the druggist had been accurate in its preparation was a matter of doubt. He believed that ordinary drug clerks were hardly competent to dispense such active medicines in pills or powders, and, therefore, when granules, prepared by experienced pharmaceutists, could not be obtained, strychnia should be given in solution, notwithstanding its intense bitterness. With regard to the dose, standard authors vary from one-thirty-secondth to one-eighth of a grain, but they are not always safe to follow. He believed one-sixteenth to be too large a dose to begin with, and should not have employed it in this case had he not known the patient's previous history; even though he had himself taken one-sixteenth of a grain three times daily without injurious results. He had in mind a patient suffering from hemiplegia, who for several weeks took about one-half of a grain three times a day, but finally had severe convulsions, which immediately subsided when the medicine was suspended. His preceptor once saw a lady who evidently died from the effects of strychnia administered in doses of one-twentieth of a grain three or four times daily for several days. He believed this remedy possessed a cumulative action. That is, while a given dose might be taken for a considerable time without ill effects, exactly the same dose might prove toxic. By this he did not mean the same dose from the bottom of a bottle containing a solution of strychnia which might be much stronger than that taken from the top; but the same dose of the medicine itself. His experience with the valerianate of zinc in this case was purely homœopathic, but to him it suggested another caution against jumping at conclusions with regard to the action of medicine. If the patient had taken the medicine before the convulsions ceased, the one out of ten who presumed to doubt its effects would, at least, have been thought presumptuous.

Dr. C. M. Fitch thought a patient who had once taken an overdose of strychnia remained more susceptible to it for a long time. He had given a patient, who had once had an overdose, one-sixtieth of a grain three times a day, and after a few doses similar effects were produced. Dr. Quine had not been a believer in the cumulative action of medicines, and thought the trouble in most cases was brought about by giving medicines faster than they were eliminated. He thought this was true of strychnia. He agreed with Dr. Fitch in reference to increased susceptibility. Dr. Pierson had given strychnia to a patient who had become alarmed and presented symptoms of poisoning, but when he subsequently gave it in the same dose disguised, it had had no bad effects. Dr. Strong had once given a patient with Bright's disease two doses of strychnia of one-thirty-secondth of a grain each, and the last dose was soon followed by convulsions simulating those produced by it. The patient died on the next day. He was not sure whether the convulsions resulted from the medicine or uræmia. Dr. Taggart had often prescribed strychnia in one-sixteenth of a grain doses, but had not seen any ill effects from it. Dr. Earle had recently seen a patient, a hard drinker, who, with suicidal intent, had taken four hundred grains of chloral hydrate at once, without the desired effect. The following night he took what was purchased for ten grains of sulphate of morphine, and made another failure. The patient had not been addicted to the use of opium, but as the chloral did not kill him, the doctor thought he had taken ten grains of morphia with impunity. Dr. Knox once prescribed four hundred and eighty grains of chloral in solution for a case of delirium tremens, and the patient had taken it all in one night without any apparent ill effects. He thought the alcohol antagonized its action. Dr. Earle reported a case of post pharyngeal abscess, consequent upon scarlet fever.

Dr. Fitch presented a specimen of polypoid tumor of the uterus, which he had removed from the patient of another physician by means of the galvano cautery. The tumor began to appear about one year ago, and the patient had since suffered at times from excessive and well-nigh fatal hæmorrhage. The tumor was attached high up in the cervical canal by means of a short, thick pedicle, and at the time of its removal it was nearly as large as a goose's egg, and of a dark red color. The loop of wire was passed around the pedicle of the tumor, and heated by means of nine large-sized Bunsen cells. The incision was as smooth as if it had been made with a knife. No hæmorrhage followed, and the patient went on to a rapid recovery. After some discussion as to the nature of the tumor, the Society adjourned.

