# Correction

**Published:** 1874-08-01

**Authors:** E. F. Ingals

**Affiliations:** 34 Throop St., Chicago


EDITOR OF THE MEDICAL EXAMINER:

*Dear Doctor:* Will you please correct an important misprint, which occurs in your issue of July 15th, 1874, page 353, line 13? I am there made to state that a certain patient took one-half a grain of sulphate of strychnia three times a day, etc. It should have read, one twentieth of a grain.

I ask this correction for fear that some one, with more faith in drugs than judgment in their use, may be tempted to try this extraordinary dose.

Yours truly,

E. F. INGALS, M.D.,

34 Throop St., Chicago.

July 19, 1874.

